# Inspiring change: humanities and social science insights into the experience and management of breathlessness

**DOI:** 10.1097/SPC.0000000000000221

**Published:** 2016-06-29

**Authors:** Rebecca Oxley, Jane Macnaughton

**Affiliations:** a Department of Anthropology, Centre for Medical Humanities; b Centre for Medical Humanities, School of Medicine, Pharmacy and Health, Durham University, Durham, England, UK

**Keywords:** breathlessness, culture, dyspnoea, language, multidisciplinary research, patient experience

## Abstract

**Purpose of review:**

Breathlessness can be debilitating for those with chronic conditions, requiring continual management. Yet, the meaning of breathlessness for those who live with it is poorly understood in respect of its subjective, cultural, and experiential significance. This article discusses a number of current issues in understanding the experience of breathlessness.

**Recent findings:**

Effective communication concerning the experience of breathlessness is crucial for diagnosis, to identify appropriate treatment, and to provide patients with the capacity to self-manage their condition. However, there is an evident disconnect between the way breathlessness is understood between clinical and lay perspectives, in terms of awareness of breathlessness, the way symptoms are expressed, and acknowledgement of how it affects the daily lives of patients.

**Summary:**

The review highlights the need for integrated multidisciplinary work on breathlessness, and suggests that effective understanding and management of breathlessness considers its wider subjective and social significance.

## INTRODUCTION

Breathing is a physiological universal, but also a subjective and socially mediated experience. Ways of breathing can identify states of being: contemplation, exertion, respiratory health, or disease. In certain chronic conditions, dyspnoea – or breathlessness in lay terms – often emerges as the most immediate and present symptom, requiring continual management. Yet, the question arises of what we mean by breathlessness. How does it feel to be breathless, and how can we communicate its experience? These questions are crucial in a context where dyspnoea (the pathological term for breathlessness) affects over 10% of the general population (with higher prevalence in specific groups) [1], and considerably impacts quality of life, but is poorly understood in terms of its subjective, cultural, and phenomenological (experiential) significance. This study follows key issues concerning the management of dyspnoea, describing the implications and potential improvements that a multidisciplinary focus, taking in insights from the humanities and social science, and might bring through seeking to comprehend breathlessness as it affects the rhythm of daily life for those that live with it. 

**Box 1:**
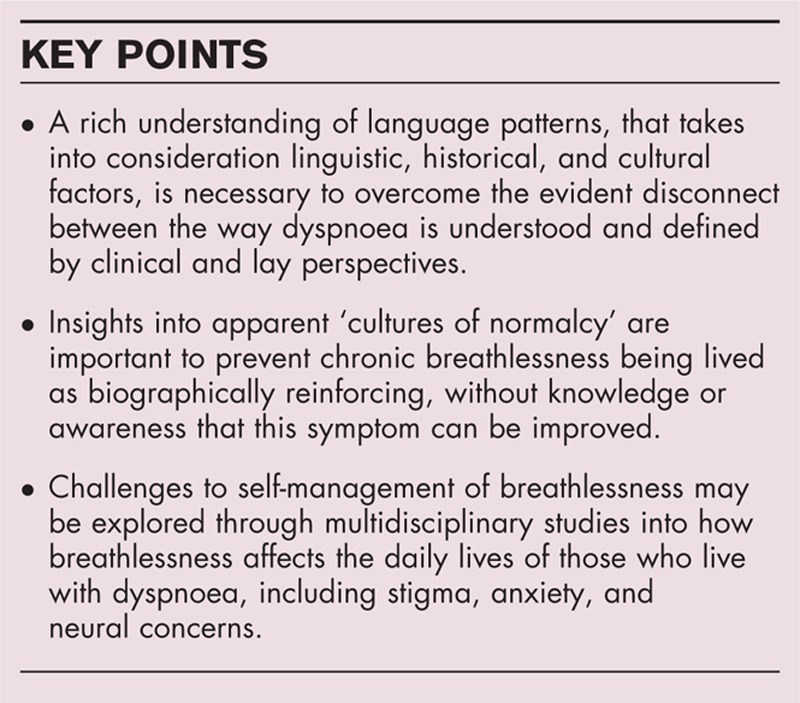
no caption available

## THE LANGUAGE OF BREATHLESSNESS

Chronic breathlessness can be life-changing; the unwelcome sensations of breathlessness and its affects may be evoked constantly with every movement, activity, and decision. This has been poignantly explored in philosopher Carel's [2^▪▪^] book, *Illness*, and is often the reason for initial clinical consultation. Dyspnoea is recognized as the ‘first vital symptom’ of respiratory illness [3,4], and thus effective communication during the clinical encounter is central to diagnosis, but also to identify appropriate treatment and provide patients with the capacity to understand and self-manage their condition. Yet, there is an evident disconnect between the way breathlessness is understood, assessed, and defined between clinical and lay perspectives. This disjunction is clear in research that describes how the measurement of dyspnoea, in terms of its neurophysiological properties, and of lung capacity through spirometry, are not always aligned to the intensity and discomfort of breathlessness, nor how it is lived on a day-to-day basis [5^▪^,^6^]. It is similarly indicative of the ‘language of dyspnoea’ [7^▪^], or the very words used to explain and express breathlessness. For example, breathlessness may not always be identified by patients to be their primary complaint; instead, it might be a change to personal routines, habits, and livelihood. Although dyspnoea has been defined as ‘a subjective experience of breathing discomfort that consists of qualitatively distinct sensations that vary in intensity’ [8], for those living with it, it might mean an inability to dance anymore [9]. There seems an uneasy tension between the personal, often highly emotive language used by patients with that used by healthcare professionals. Such variations in description can affect recognition of breathlessness and/or underlying medical conditions, and call for a further understanding of the subjective ways dyspnoea is conceived of.

Dyspnoea has been recognized as a multidimensional construct [10], with common sensations recognized to include ‘air hunger’, ‘the effort of breathing’, and ‘chest tightness’ [8], although associations between such sensations continue to be explored [11,12]. Such research can be important to shape comprehension of disease aetiology [13], yet the narratives of patients and carers do not often fit neatly into such categories. Studies of breathlessness in Uruguay and Mexico have noted that particular attention should be given to the metaphors used to express it, and that cultural phrases and terms can be used interchangeably to describe sensations [14,15]. Phrases, furthermore, may not have an equivalent in or translate to English language or clinical terms. For instance, ‘agitation’, which is commonly identified by Mexican-Spanish chronic obstructive pulmonary disease (COPD) patients to express their sensations of dyspnoea, does not have a corresponding term in English [15], nor does ‘air hunger’ easily translate to Spanish [15]. However, a rich understanding of language patterns could offer the potential for differentiation between different experiences of breathlessness and the underlying conditions of dyspnoea. Studies have found that clusters of descriptors – including those based on emotive language expressing the intensity and distress of dyspnoea – could mediate pathophysiological conditions, but that cultural, socioeconomic, linguistic, and educational backgrounds influence the use of particular terms [7^▪^,^16^,^17^].

Another key study has explored how ethnicity can influence ways to describe (induced) breathlessness [18^▪▪^]. Hardie *et al.* found that African-American people tended to use upper airway descriptors to convey their feelings of discomfort, whereas those who identified as white used words that suggested chest wall sensation. As the phenomenological philosopher Merleau-Ponty [19] once declared, ‘The spoken word is a gesture, and its meaning the world’ (p. 184), and these studies certainly point to the influence that patients’ backgrounds and environment can have on the experience of breathlessness: where language of breathlessness does not only express bodily sensation but also speaks of cultural reality. Thus, a socially appropriate analysis of language is necessary when seeking to interpret sensations and report symptoms, and cultural descriptors and traditional metaphors or phrases are key tools in this process.

The vital nature of a contextual understanding of breathlessness is also evident when investigating a singular expression: what do we mean by the term ‘wheeze’? Medically, a wheeze has a specific meaning: a high pitched, whistling lung sound generated at the end of inspiration, or early expiration through compressed airways [20]. However, the experience of wheezing can be interpreted quite differently by those who live with it, and the meaning of ‘wheeze’ as a medical term can be intrinsically subjective. Research on young people with asthma and their caregivers found that caregivers considered a wheeze was something to heard, whereas for young people, it was something that was felt, as a wheeze has sensation [21]. In this sense, the term can be descriptively limiting in clinical situations, also considering that what physicians understand as a ‘wheeze’ can be described through other expressions, most commonly ‘cough’, ‘rattle’, or simply asthma [22]. These words have been noted to be highly figurative and etymologically distinct [23^▪▪^], and given this, it is not surprising that studies have found less than a 30–50% consensus between clinical understandings of wheeze with those of parents of asthmatic children [24,25]. Such conceptual disjunctions of what ‘wheeze’ means can lead to a miscommunication of the prevalence of wheeze in patient experience [24,26], or the severity of this symptom and perceptions of control [27,28]. Following on from this, the need is evident for a greater linguistic, historical comprehension of respiratory phrases and experiences, and this is something that has been stressed in the literature [23^▪▪^]. It is apparent that working with linguistics, looking at figurative language, and using corpus linguistics (the study of language as expressed in real world ‘texts’ such as patient accounts) [29] would be very valuable in illuminating what it is people mean by their breathlessness.

Disparities in the language of dyspnoea could imply the need for greater popular health literacy – a push to educate and promote medical understandings of ‘wheeze’ (and wider terms of breathlessness) to patients, carers, and the wider community. Indeed, those with greater health literacy may be more equipped to manage their disease [30], picking up on the notion of the ‘expert patient’ who has ‘the confidence, skills, information, and knowledge’ to manage their illness effectively [31]. However, there is another, perhaps more beneficial approach – one which is arguably already encouraged, if underdeveloped – which is to acknowledge variations in language and experience, and explore these, working with patients to uncover their experiences of breathlessness in their own terms. With wider multidisciplinary insights from the medical humanities in conjunction with the health sciences, we might become more aware of what breathlessness means, how it is expressed, and how to provide effective care for those with even the lowest health literacy – often those from communities that live with high rates of dyspnoea, where breathlessness may be a ‘normal illness’ or way of life [32].

## AWARENESS OF BREATHLESSNESS

Public awareness of breathlessness as a health concern is imperative for early and accurate diagnoses of chronic conditions as well as prevention. This is complicated by the fact that breathlessness often remains invisible within certain communities, where there is ‘lack of a public story’ for respiratory illnesses such as COPD [33^▪^]. Dyspnoea has been well explored through qualitative research as being a perpetual reminder of disease, an ‘interruption’ to normal life, and ‘biographically disruptive’ [34–36]. This involves, for patients and carers, an uneasy focus of attention on the breath, which is not typically brought to consciousness. This brings about disruptions to everyday life assumptions and practices, such as the ability to exercise, concentrate, and cope with distressing acute exacerbations, and associated disruptions to personal conceptions of identity [37–39]. Crucially, there is a mobilization of resources to deal with this sensation, as breathlessness becomes something to be treated and self-managed. Yet, a model of disruption, common to understand chronic illness, does not always explain the experiences of those with debilitating breathlessness, including those who do not seek help – such as the estimated two million people with undiagnosed COPD in the United Kingdom [40].

Here, we might explore the reasoning behind these experiences and numbers also by looking to the concept of awareness, because for many, chronic breathlessness is not life-changing, nor eventful; the flow of daily life remains unchanged. Leder [41], a prominent anthropologist, promotes becoming aware of bodily sensations as ‘dys-appearance’, or dysfunctional appearance, but here, awareness of breathlessness recedes into the rhythm of what is considered usual or regular; breathlessness becomes absent to conscious attention once more. Certainly, for many of those living with COPD, the slow progression of disease means that breathlessness may provide a sense of continuity, with disruption paradoxically becoming a way of life and providing intense suffering only when breathlessness rises to attention during an acute exacerbation [42]. In certain contexts – often those in lower socioeconomic regions, where smoking is prevalent – there appears to be a ‘culture of normalcy’ of breathlessness, where it can be distressing and debilitating, but is not perceived as disruptive to one's sense of being. Instead, breathlessness is taken for granted, or expected as a common experience of the ageing process, and does not impact individual and community life [32,42,43]. Insights into the normalizing culture, and appreciation of the subjective approach to living with dyspnoea are critical to prevent breathlessness being lived as biographically ‘reinforcing’ [44], with everyday suffering being further perpetuated without knowledge or awareness that this symptom can be improved.

## BREATHLESSNESS AFFECTS THE RHYTHM OF DAILY LIFE

Self-management is an important factor in living well with breathlessness. Disease-specific self-management of dyspnoea has been proven to provide significant benefits to patients’ well-being and sense of control over their illness, along with economic benefits [45,46]. Pulmonary rehabilitation as a programme has particularly been lauded for improvements in quality of life, mortality rates, and exercise capacity [47]. However, there are continuing challenges regarding levels of pulmonary rehabilitation uptake and continued exercise and activity [48]. Research has begun to tease out how chronic breathlessness affects the daily lives of those that suffer from it, which might make visible or more evident certain barriers to effective self-management.

Qualitative literature suggests that ‘pacing’ has become more than an overt management approach (involving paced exercise and breathing) for patients, but has become a way of life. Pacing involves constant acknowledgement of personal limits in terms of mental and physical capacity, along with calculated navigation of surroundings, including air quality, temperature, and physical environment to avoid exacerbation [49]. Owing to this, practical infrastructure, including transport and location of healthcare services remains a great concern to many patients [50], once more emphasizing the need for services to be easily accessible and strategically located to benefit patients. Pacing also involves a careful balancing act of surrendering personal control, and accepting limits of dependency, while attempting to maintain independence [51]. This can be a struggle for many living with breathlessness, considering that ‘pacing’ can take place in the context of a shrinking lifeworld where lifestyle changes provide a sense of increasingly bounded and constrained space [49,52]. Not only can there be physical signs of contraction, such as with the changing of bodily posture and weight in COPD patients, but there is also apparent shrinking of resilience, stamina, social interaction, and physical as well as cognitive ability [49,53]. For those with chronic dyspnoea, there is ultimately a sense of the diminishment of identity; of not being able to be the person that they once were or wish to be [54]. Such experiences of pacing and shrinking result in people having ‘safe spaces’, or areas where they perceive they can manage their breathlessness more comfortably without worry of stigma, or fear of an acute episode.

Accounts of breathlessness in fiction capture this sense of the pacing and rhythm of breathlessness in ways that may be well recognized by patients, but also explores the influence on the body of the external environment. Roberts’ [55] novel, *Breath,* describes the experience of a man awaiting a lung transplant having been injured in a gas attack: ‘Baras closes his eyes and tries to settle his breath into a slower, deeper rhythm. Ever since his lungs were damaged, he has found it hard to see it as a failure of his own body. Somehow, even now on the brink of having his weakest lung cut out and replaced with a new one, he can’t locate the problem in his own chest […] it still feels like a problem with the air, not his own body’ (p. 103).

Stigma remains a key factor in the experience of dyspnoea, and self-stigmatization, through feeling unworthy of care, has been recognized as one of the major factors of not attending pulmonary rehabilitation after an acute episode for those with COPD [56^▪^]. Greater understanding of the impact of stigma on well-being, and access to care will be critical to overcome barriers to self-management, but also further inquiry into how patients negotiate wider ideologies surrounding dyspnoea and/or respiratory disease. Narrative analysis has already shown that the beliefs of those with COPD do not always align to clinical knowledge, including scepticism that smoking could have contributed to subjective experiences of illness [57]. Such accounts have been linked to a movement away from self-blame for illness, to maintain a personal sense of identity and integrity [58], which has practical implications as this might allow for continued motivation and health-seeking behaviour.

Emotional states have been found to correlate strongly to self-management behaviour in those with COPD [59], so it is little wonder that the anticipation of stigma as well as increased breathlessness outside of ‘safe spaces’ is recognized as a barrier to effective care. Promising research looks at how to break the anxiety–dyspnoea cycle, which has a severe impact on patients’ livelihoods as well as number of hospital admissions [60], through mindfulness and/or cognitive behavioural therapy. Qualitative and quantitative studies have found mindfulness to be beneficial in reducing feelings of anxiety and improving quality of life for COPD sufferers, although further work is needed to draw firm conclusions [61^▪^,^62^]. Early indications also promote the use of psychological activity in pulmonary rehabilitation [63]. This is somewhat supported by research from Williams *et al.*[64^▪▪^] looking into the experience of activity for COPD sufferers. This study concludes that psychosocial and physical movement along with the benefits of being exposed to fresh air brings meaning into the lives of those with COPD – rather than a sense of stagnation. It proposes that individualized exercise activities outside the confines of a gymnasium – where pulmonary rehabilitation is generally situated – may be more valued and constructive. Wider research, however, still endorses the benefits of a comprehensive and intensive intervention programme, but acknowledges that this may not align with the way that breathlessness is lived day-to-day [49].

More work is thus required to understand the complex and subjective ways that breathlessness as a biopsychosocial experience is lived on a daily basis by those with chronic dyspnoea, particularly when considering approaches to encourage self-management. In doing so, it must be explored in ways that value a range of perspectives, and particularly those of people who live with chronic breathlessness. As Torheim *et al.*[65] have indicated, feeling recognized, and listened to is important for improving the respiratory illness experience. Acknowledgement can come in a range of forms, however, and one further strand of research that offers hope to comprehend this complexity is neuroscience, given that the correlation of cognition, affect, and breathlessness is one which underpins the way patients perceive their lifeworld and illness. This approach is gaining momentum as technological advances allow for more thorough inquiry, via MRI imagery, to understand not only cognitive decline and structural change in neural matter for those who live with chronic breathlessness [66,67^▪^] but also how this impacts their experience of dyspnoea as distressful.

## CONCLUSION

Living well with chronic breathlessness is important not only for patients, carers, and their families, but also for clinicians, service managers, and allied health professionals. The successful understanding of dyspnoea, in terms of its expression, sensation, communication, social mediation, and subjective experience is an implicitly shared goal. In this sense, a multidisciplinary approach to breathlessness that takes into account its subjective, cultural, and linguistic – as well as medical – significance is paramount to increasing the awareness and visibility of an arguably invisible experience. Perhaps, if the meaning of breathlessness is taken as a primary concern, we can gain key insights of how to improve the ways dyspnoea is perceived and managed by those who live with it.

## Acknowledgements

We would like to gratefully acknowledge our Life of Breath colleagues for their engaging conversations and valuable insights, particularly Andrew Russell, Arthur Rose, Mary Robson, Corinne Saunders, and to Sarah McLusky for her guidance and support.

### Financial support and sponsorship

The work was supported by the Life of Breath Project, funded by the Wellcome Trust [grant number 103339], Centre for Medical Humanities, Durham University, and Department of Philosophy, the University of Bristol.

### Conflicts of interest

There are no conflicts of interest.

## REFERENCES AND RECOMMENDED READING

Papers of particular interest, published within the annual period of review, have been highlighted as:

▪ of special interest
▪▪ of outstanding interest
